# Atypical Lipomatous Tumor/Well-Differentiated Liposarcoma with Intramuscular Lipoma-Like Component of the Thigh

**DOI:** 10.1155/2020/8846932

**Published:** 2020-12-12

**Authors:** Chairat Burusapat, Nuttadon Wongprakob, Nutthapong Wanichjaroen, Chatchai Pruksapong, Kantang Satayasoontorn

**Affiliations:** ^1^Division of Plastic and Reconstructive Surgery, Department of Surgery, Phramongkutklao Hospital and Phramongkutklao College of Medicine, Bangkok, Thailand; ^2^Department of Pathology and Laboratory Medicine, Phramongkutklao Hospital, Bangkok, Thailand

## Abstract

Atypical lipomatous tumor/well-differentiated liposarcoma (ALT/WDLPS) is a locally aggressive mesenchymal neoplasm composed either entirely or partly of an adipocytic proliferation showing at least focal nuclear atypia in both adipocytes and stromal cells. ALT most frequently occurs in deep soft tissue of proximal extremities (thigh and buttock) and usually presents as a deep-seated, painless mass that can slowly attain a very large size, which is one of the most common sarcomas of extremity. In the patients who presented with deep-seated tumor, distinction between intramuscular lipoma and well-differentiated liposarcoma is important due to the differences in treatment and prognosis. However, atypical well-differentiated liposarcoma with intramuscular lipoma-like component of the thigh is extremely rare. Moreover, the infiltrative growth pattern or intramuscular component may lead to a misinterpretation as intramuscular lipoma on a small biopsy. We present an unusual case of a female who presented with symptomatic mass at the thigh which has rarely been reported in English literature as an infiltrative intramuscular lipoma-like growth pattern of well-differentiated liposarcoma. Therefore, preoperative diagnosis is necessary, and correlation with imaging studies is required when one encounters with a large deep-seated mass. Atypical lipomatous tumor or well-differentiated liposarcoma should be kept in mind in the patient who presents with abnormal thigh mass.

## 1. Introduction

Atypical lipomatous tumor/well-differentiated liposarcoma (ALT/WDLPS) is a locally aggressive mesenchymal neoplasm composed either entirely or partly of an adipocytic proliferation showing at least focal nuclear atypia in both adipocytes and stromal cells. “Atypical lipomatous tumor” and “well-differentiated liposarcoma” are synonyms describing lesions that are morphologically and genetically identical. Amplification of *MDM2* and/or *CDK4* is almost always present. ALT/WDLPS represents the largest subgroup of adipocytic malignancies, accounting for approximately 40-45% of all liposarcomas. These lesions occur predominantly in middle-aged adults, with peak incidence between the fourth and fifth decades of life. ALT most frequently occurs in deep soft tissue of proximal extremities (thigh and buttock) and usually presents as a deep-seated, painless mass that can slowly attain a very large size [1]. In the patients who presented with deep-seated tumor, distinction of intramuscular lipoma and ALT/WDLPS is important due to the different treatments and prognosis. While ALT/WDLPS usually consists of a large, well-circumscribed, lobulated mass, some cases have partial replacement of surrounding muscle tissue with entrapped striated muscle fibers resembling intramuscular lipoma. Generally, the presence of infiltrative margins and intermingled muscle fibers in intramuscular lipoma are a characteristic and indicates a benign lesion rather than malignancy on magnetic resonance imaging [2, 3]. This component of ALT/WDLPS may cause confusion with intramuscular lipoma on histologic examination and lead to a misdiagnosis. Careful sampling of these tumors is mandatory because portions of an intramuscular atypical lipomatous neoplasm may be indistinguishable from intramuscular lipoma [4].

Here, we present a rare case of ALT/WDLPS with obvious infiltrative intramuscular component which can be confused with intramuscular lipoma.

## 2. Case Report

**Figure 1:**
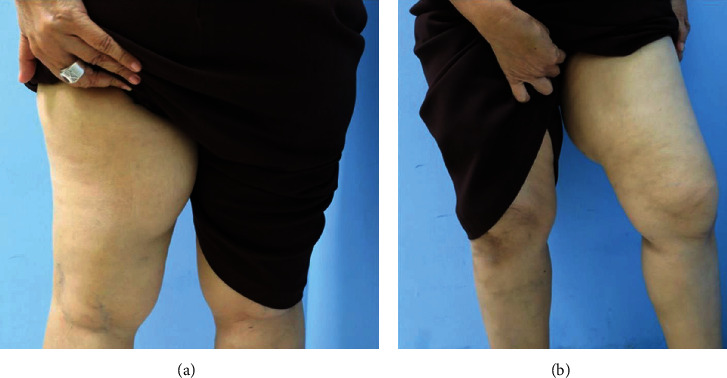
Demonstrated mass at left thigh: (a) posterior view, (b) front view.

A 61-year-old Thai woman presented with a nontender mass at the left posterior thigh rapidly growing for 6 months. She had no any underlying or previous surgery. She had pain when she walked and slightly limited her left leg function during motion. On physical examination, there was a firm nonpulsatile mass over left medial thigh measuring ~10 x 6 cm in diameter (Figure 1). There was no notable grossly skin involvement, and the mass appears not fixed to bony structure. No sensory deficit is identified. The left inguinal lymph node cannot be palpated.

**Figure 2:**
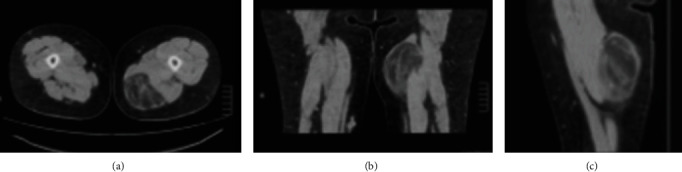
Computed tomography (CT) scan demonstrated 11 x 8 x 7 cm, in vertical x transverse x AP diameter of well-defined, fatty lesion with thick streak fibrous tissue at the right posteromedial of thigh: (a) axial view, (b) coronal view, and (c) sagittal view.

**Figure 3:**
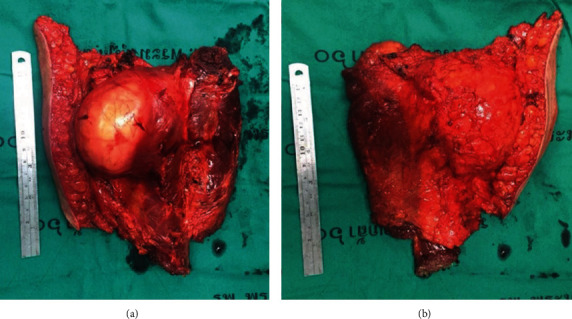
Complete surgical removal of tumor.

**Figure 4:**
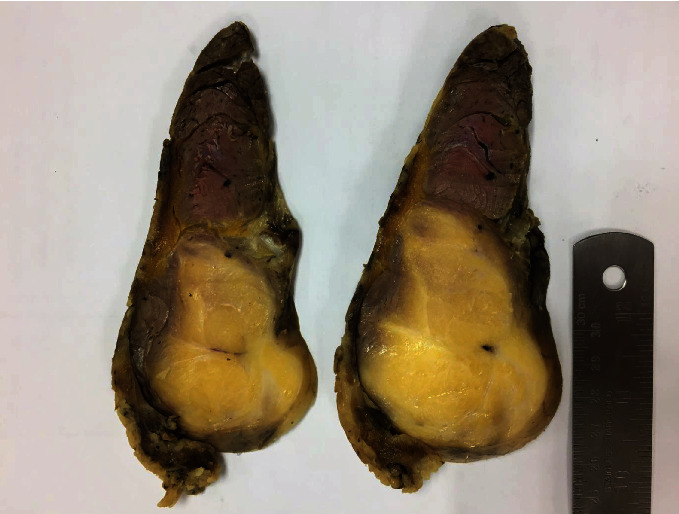
The gross specimen showed cut surfaces of the mass revealing pale yellow, greasy, lobulated mass with ill-defined, infiltrative edge to the surrounding muscle on one side.

**Figure 5:**
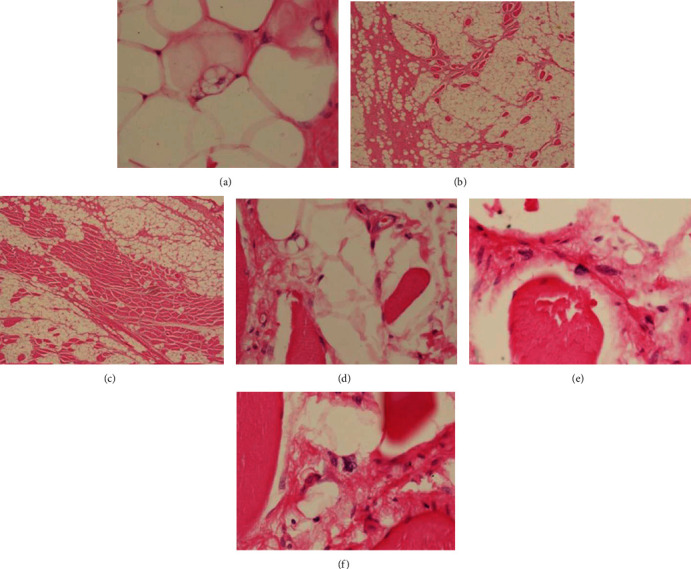
Microscopic examination. (a) Histological revealed characteristic multivacuolated lipoblasts. (b, c) An infiltrative, intramuscular component is composed of adipocytic lesion encasing skeletal muscle fibers. (d–f) Multivacuolated lipoblast and hyperchromatic stromal cells found in between the muscle fibers.

The computed tomography (CT) scan demonstrated an intramuscular 11 x 8 x 7 cm, in vertical x transverse x AP diameter of well-defined, fatty lesion with thin streak fibrous tissue at the left posteromedial of the thigh (Figure 2). Fine needle biopsy (FNA) suggested lipoma but liposarcoma cannot be excluded. Then, the core needle biopsy was performed and showed well-differentiated liposarcoma. Metastasis workups were unremarkable. The operation was performed on supine position under general anesthesia. Vertical incision was performed. The tumor was confined in the semimembranosus and semitendinosus muscle. Complete en bloc tumor resection with total removal of semimembranosus and semitendinosus muscles was successful (Figure 3). The gross specimen consisted an intramuscular mass, measuring 20x17x6 cm with yellow, lobulated, rubbery, rather well-circumscribed cut surfaces. The mass is generally well delineated from the surrounding tissues by a thin white translucent, glistening membrane. Serial sections of the mass revealed focal gradual replacement of the muscle tissue by fat into the intermuscular connective tissue spaces (Figure 4). The other areas showed yellow smooth rubbery cut surface. Microscopic examination showed an adipocytic neoplasm. The tumor was mainly composed of small and large adipocytes with interspersed fibrous septa that contain large, irregular, hyperchromatic nuclei. Small nucleoli presented in some of the large cells. There were scattered atypical cells containing large hyperchromatic nuclei in collagenous background. Intramuscular component resembling intramuscular lipoma at periphery of the mass was also presented characterized by adipocytes diffusely infiltrating muscle. The entrapped muscle fibers usually show few changes other than various degrees of muscular atrophy. There were also few lipoblasts and stromal cells with atypical nuclei in fibrous septa between muscle fibers as seen in ALT/WDLPS present. No necrosis and no high-grade sarcomatous component were seen (Figure 5). The final pathologic diagnosis was well-differentiated liposarcoma with intramuscular lipoma-like component.

## 3. Discussion

Well-differentiated liposarcoma is the most common histologic subtype of liposarcoma, accounting for 40% to 45% of all liposarcoma therefore representing the larger subgroup of adipocytic malignancies. Well-differentiated liposarcoma is subdivided in the adipocytic (lipoma-like), sclerosing, inflammatory, and spindle cell subtypes, of which the adipocytic-like and sclerosing-type are common [5].

Adipocytic well-differentiated liposarcoma is composed of a relatively mature adipocytic proliferation, featuring cell size variation as well as at least focal nuclear atypia. Varying number (from many to none) of lipoblasts may be found [5]. However, an infiltrative growth pattern or intramuscular component can be rarely seen in this reported case. The infiltrative growth pattern or intramuscular component may lead to a misinterpretation as intramuscular lipoma on a small biopsy. Therefore, correlation with imaging studies is required when one encounter with a large deep-seated mass.

Distinguishing between intramuscular lipoma and well-differentiated liposarcoma can be difficult based on histologic analysis alone. Because of differences in treatment, prognosis, and long-term follow-up, it is important to preoperatively distinguish simple lipoma from well-differentiated liposarcoma [6]. Correct classification is important, because aggressive local disease recurrence occurs more frequently in patients with well-differentiated liposarcoma than in patients with intramuscular lipoma [7].

Computed tomography (CT) and magnetic resonance imaging (MRI) have been used for preoperative identify between intramuscular lipoma and well-differentiated liposarcoma. CT and MRI revealed fatty lesions containing streaky structures in benign lesions. In well-differentiated liposarcoma, thick streaks represented entrapped muscle fibers, and thin streaks represented fibrous tissue or neoplastic spindle cell proliferation. Furthermore, CT in well-differentiated liposarcoma revealed foci of hazy amorphous density, representing spindle cell proliferation [8]. As for the well-differentiated liposarcoma, thick septa and nodular or patchy nonadipose components were present more frequently in deep lesions than in subcutaneous lesions. The septa in well-differentiated liposarcoma enhanced more strongly than in benign lipoma. The septa showed no enhancement relative to muscle in benign lipoma, whereas the septa showed moderate or marked enhancement in all well-differentiated liposarcoma [9].

Moreover, MRI revealed significantly enhanced signal in well-differentiated liposarcoma in a background of multiple well-differentiated benign fatty masses by showing the increased vascularity in the septa of well-differentiated liposarcoma. Although such signal enhancement can be seen in some types of benign lipomatous tumors with increased blood vessels, this technique is helpful in selection of biopsy site, especially in a clinical setting of multiple fatty masses [10].

When the data for solitary lipoma were compared to soft-tissue sarcoma, it was found that patient age and duration of symptoms were of minor value in the clinical differential diagnosis. However, if a tumor was larger than 5 cm, irrespective of depth and location, located in the thigh, irrespective of depth and size, or deep, irrespective of location and size, it was more likely to be a sarcoma [10].

Patients with extremity tumors had a significantly better prognosis than those with retroperitoneal or scrotal tumors. Extremity tumors treated by wide local excision recurred in only 11% of cases, whereas 60% of those treated by marginal or simple excision recurred [11]. Although, metastases and deaths due to tumor had never been reported, Evans et al. reported 17% suffered inoperable recurrence and 10% died as a result of the neoplasm [12].

Intramuscular lipomas were smaller than well-differentiated liposarcoma, but there was significant overlap between the 2 groups. Four percent of patients with intramuscular lipoma and 27% of patients with well-differentiated liposarcoma developed local disease recurrence [7]. Disease recurrence did not correlate with patient age at diagnosis, gender, tumor size, and tumor location.

A well-differentiated liposarcoma of any type has no potential for metastasis unless they undergo dedifferentiation [5]. Occasionally, the terms atypical lipoma, atypical intramuscular lipoma, and well-differentiated liposarcoma made the plastic surgeon confused. Well-differentiated liposarcoma and atypical lipoma should be considered synonyms, and their use should therefore be determined by the degree of reciprocal comprehension between the surgeon and the pathologist to prevent either inadequate or excessive treatment.

Generally, when soft tissue tumor exists throughout the body in large sizes, if deeply located and infiltrating, a diagnosis of a soft tissue sarcoma must be considered [13]. Positive resection margins were associated with reduced local recurrence-free survival [14].

A review of the literature showed only two cases of retroperitoneal liposarcoma associated with multiple subcutaneous lipomas, two cases of liposarcoma involving an extremity associated with multiple subcutaneous lipomas, and one case of intramuscular liposarcoma associated with multiple intramuscular lipomas [15].

## 4. Conclusion

Atypical well-differentiated liposarcoma with intramuscular lipoma-like component of the thigh is extremely rare. The infiltrative growth pattern or intramuscular component may lead to a misinterpretation as intramuscular lipoma on a small biopsy. Therefore, preoperative diagnosis is necessary and correlation with imaging studies is required when one encounter with a large deep-seated mass. Atypical lipomatous tumor or well-differentiated liposarcoma should be kept in mind in the patient who presents with abnormal thigh mass.
